# 

**Published:** 1888-01

**Authors:** 


CONTENTS

Vol. 35 No. 1.

| | PAGE. |
|---|---|
| The New Year | 1 |
| Some Seasonable Hints | 3 |
| Temporenzia | 7 |
| Dogbury Down on the Rubbers | 8 |
| The Cocoanut Tree | 9 |
| Marriage is a Partnership | 10 |
| A Dream Realized | 11 |
| Preservation of Health | 13 |
| A Case in Point | 13 |
| The Spleen, its Functions | 14 |
| Food for Young and Old | 15 |
| The Means of Contagion in Scarlet Fever | 17 |
| Burial Reform Association | 17 |
| Book Notices | 18 |
| Household | 19 |
| Miscellaneous | 22 |

INDEX TO ADVERTISEMENTS OF HALF A PAGE AND OVER.

| | PAGE. |
|---|---|
| Crystal Pepsin Tablets | I |
| Hollow Suppositories | II |
| Before it is Born | III |
| Crystalline Phosphates | IV |
| Lactopeptine | V |
| Castoria | V |
| On the Wheel | VI |
| Bovinine | VII |
| Telegraph Medicine Co. | VIII |
| Dr. Molesworth | VIII |
| J. B. Watkins' Land Mortgage Co. | VIII |
| Celerina | X |
| Winchester's Hypophosphates | XI |
| Zylonite | XI |

